# Chemotherapy combined with radiotherapy can benefit more unresectable HCC patients with portal and/or hepatic vein invasion: a retrospective analysis of the SEER database

**DOI:** 10.3389/fonc.2023.1098686

**Published:** 2023-06-20

**Authors:** Xiaotong Qiu, Jianye Cai, Haitian Chen, Jia Yao, Cuicui Xiao, Rong Li, Jiaqi Xiao, Jiebin Zhang, Xin Sui, Tongyu Lu, Jun Zheng, Yingcai Zhang, Yang Yang

**Affiliations:** ^1^ Department of Hepatic Surgery and Liver Transplantation Center, The Third Affiliated Hospital of Sun Yat-sen University, Guangzhou, China; ^2^ Guangdong Province Key Laboratory of Liver Disease Research, Guangzhou, China; ^3^ Department of Anesthesiology, The Third Affiliated Hospital of Sun Yat-sen University, Guangzhou, China; ^4^ Surgical Intensive Care Unit, The Third Affiliated Hospital of Sun Yat-sen University, Guangzhou, China

**Keywords:** hepatocellular carcinoma, chemotherapy, radiotherapy, surveillance, epidemiology, end results database, prognosis

## Abstract

**Background:**

The purpose of this study is to evaluate the effects of chemotherapy and radiotherapy on the prognosis of unresectable HCC patients with portal and/or hepatic vein invasion.

**Methods:**

A retrospective analysis of unresectable HCC patients with portal and/or hepatic vein invasion registered in the Surveillance, Epidemiology, End Results (SEER) database was performed. The propensity score-matching (PSM) method was used to balance differences between groups. Overall survival (OS) and cancer-specific survival (CSS) were the interesting endpoints. OS was calculated from the date of diagnosis to the date of death caused by any cause or the last follow-up. CSS was defined as the interval between the date of diagnosis and date of death due only to HCC or last follow-up. OS and CSS were analyzed by using Kaplan-Meier analysis, Cox proportional hazards model, and Fine-Gray competing-risk model.

**Results:**

A total of 2,614 patients were included. 50.2% patients received chemotherapy or radiotherapy and 7.5% patients received both chemotherapy and radiotherapy. Compared to the untreated group, chemotherapy or radiotherapy (COR) (HR = 0.538, 95% CI 0.495-0.585, p < 0.001) and chemotherapy and radiotherapy (CAR) (HR = 0.371, 95% CI 0.316-0.436, p < 0.001) showed better OS. In the COR group, Cox analysis results showed AFP, tumor size, N stage and M stage were independent risk factor of OS. Competing-risk analysis results showed AFP, tumor size and M stage were independent risk factor of CSS. In the CAR group, AFP and M stage were independent risk factors of OS. Competing-risk analysis results showed M stage were independent risk factor of CSS. Kaplan Meier analysis showed chemotherapy combined with radiotherapy significantly improves OS (10.0 vs. 5.0 months, p < 0.001) and CSS (10.0 vs. 6.0 months, p = 0.006) than monotherapy.

**Conclusion:**

AFP positive and distant metastasis are the main risk factors affecting OS and CSS of unresectable HCC patients with portal and/or hepatic vein invasion. Chemotherapy combined with radiotherapy significantly improves OS and CSS of unresectable HCC patients with portal and/or hepatic vein invasion.

## 1 Introduction

Hepatocellular carcinoma (HCC) is the most common type of hepatic malignant tumor in the world, with a high mortality rate. Approximately 10% to 40% of HCC patients show macrovascular invasion at the time of initial diagnosis (1). There are two distinct types of macrovascular invasion: portal vein tumor thrombosis (PVTT) and hepatic vein tumor thrombosis (HVTT) (2). It has been reported that the incidence of macrovascular invasion is more than doubled in patients with hepatocellular carcinoma compared with cirrhotic patients without malignancy(24.4% vs 11.4%; p = 0.05) (3). HCC with macrovascular invasion is listed in advanced stage by the American Association for the Study of Liver Diseases/Barcelona-Clinic Liver Cancer (AASLD/BCLC) staging system and treatment recommendations. However, there is no consensus on the diagnosis and treatment criteria for hepatocellular carcinoma combined with PVTT/HVTT, which brings great difficulties to the selection of treatment regimens and the prediction of treatment effects.

In recent years, hepatectomy, local interventional therapy, radiotherapy, molecular targeted therapy and immunotherapy have been widely used, which is of great significance for the quality of life and prolonging survival time of some patients. Currently, Sorafenib or Lenvatinib are recommended as first-line TKI treatment for HCC combined with PVTT (4). Several studies have shown that the combination of TKI and ICI can significantly improve the survival benefits of patients with advanced HCC (5–7), so combination therapy has become the latest trend in the treatment of advanced HCC. These studies have also shown that not all HCC patients can benefit from the combination therapy, one of the reasons is likely to be some patients with primary immune resistance.

Transarterial chemoembolization (TACE) and hepatic artery infusion chemotherapy (HAIC) have become the most commonly used palliative treatments for patients with unresectable HCC and are no longer considered an absolute contraindication for patients with PVTT/HVTT- HCC (8). Moreover, advanced radiotherapy techniques have yielded encouraging results in HCC patients with PVTT/HVTT (9, 10). For all that, current studies on chemotherapy or radiotherapy in patients with concomitant PVTT/HVTT-HCC are limited to single-center, small studies, so our study aimed to use the national-scale SEER database for a retrospective analysis to investigate the effects of chemotherapy and radiotherapy regimens on the survival of patients with PVTT/HVTT- HCC.

## 2 Materials and methods

### 2.1 Data source

The SEER registry is a multicentric database for cancer research. This database contains data from 17 areas of the United States, representing roughly 28% of the total U.S. population. Patient demographics, initial cancer location, illness grade/stage, surgery, radiotherapy, chemotherapy, and survival status can all be obtained from the SEER database.

### 2.2 Patient screening

**Figure 1:**
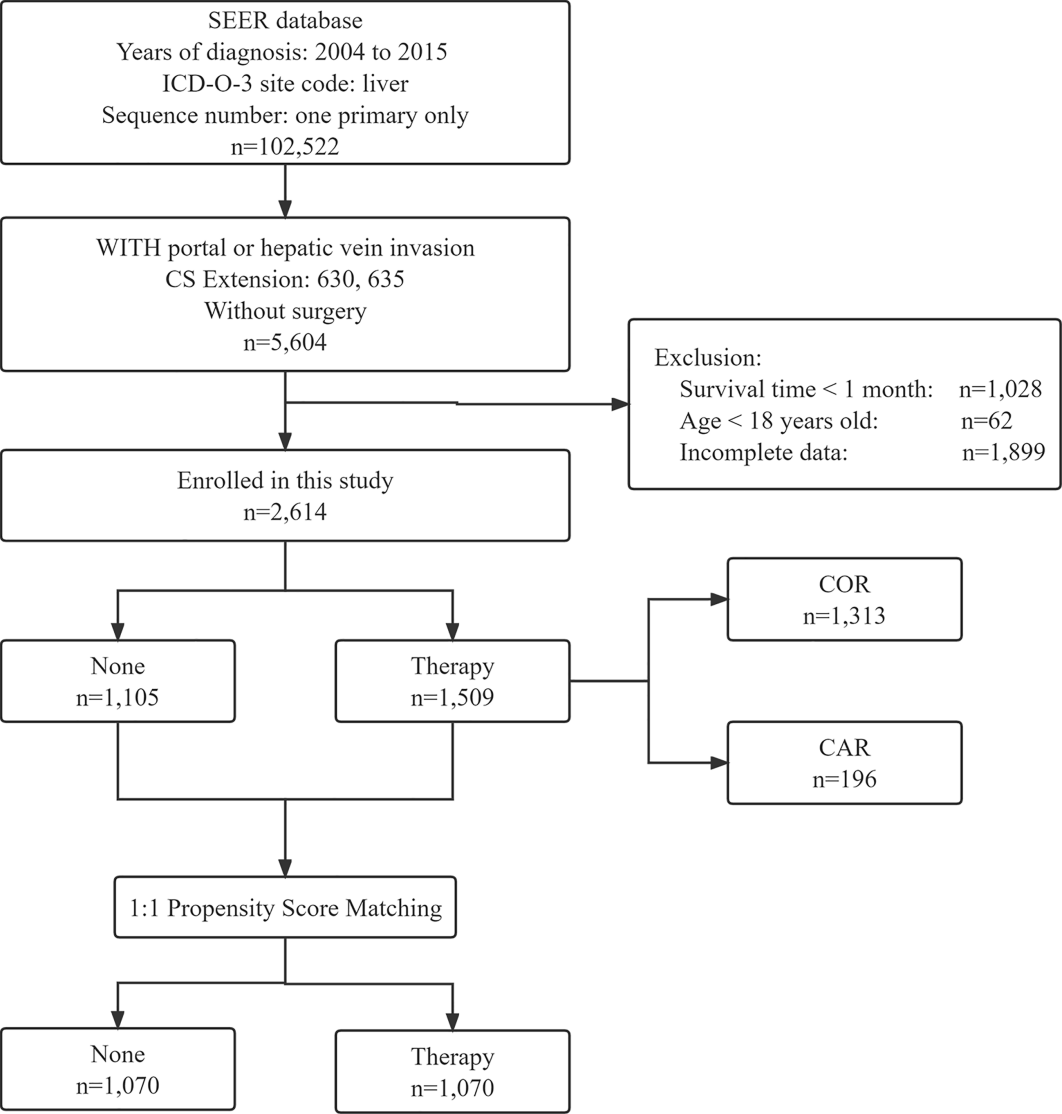
Flow chart showing the inclusion and exclusion process of patients in our study. SEER, Surveillance; Epidemiology, End Results; COR, Chemotherapy or Radiotherapy; CAR, Chemotherapy and Radiotherapy.

We use the PICO principle to filter data, P (Patient): We selected patients diagnosed with HCC from 2004 to 2015 in the SEER database according to the International Classification of Diseases for Oncology. The inclusion criteria were as follows (1): a clear histopathological diagnosis of HCC (2); the primary site of the tumor is located in the liver with only one primary tumor (3); tumor invades the portal or hepatic veins and has not been treated surgically (4); follow-up for more than one month (5); age over 18 years old (6); complete survival data. The screening process is shown in **Figure 1**. I (Intervention): We chose whether to receive radiotherapy or chemotherapy as the intervention (this was recorded as yes or no in the SEER database). C (Comparison): We compared patients who received radiotherapy or chemotherapy alone with those who received radiotherapy combined with chemotherapy. O (Outcome): Overall survival (OS) and cancer-specific survival (CSS) were the interesting endpoints. T (time): The deadline for follow-up is December 31, 2022.

### 2.3 Clinical variables of patients

Demographic variables include age, sex, race, marital status, AFP, tumor size, SEER stage (SEER-specific), N and M stage, TNM, radiotherapy, chemotherapy, SEER classification of causes of death, months of survival, and final status. Since SEER stage, M stage and TNM results are the same, we only show the results of M stage.

### 2.4 Statistical analysis

**Figure 2:**
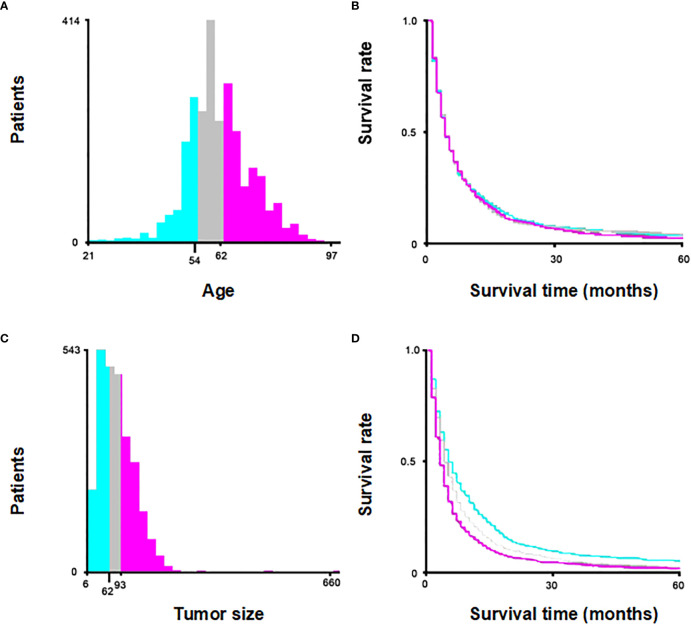
Optimal cut off value of age **(A, B)** and tumor size **(C, D)** using X-tile software.

Age and tumor size were categorically divided based on the optimal cut-off value generated by X-tile software version 3.6.1 (Yale University School of Medicine, US) (**Figure 2**). Categorical variables were expressed as a number (percent, %) and compared by the chi-square test. In order to minimize the effect of confounding factors when comparing between two groups, a 1:1 propensity score-matching (PSM) method was used to match with None and Therapy group. The nearest-neighbor matching algorithm without replacement was applied to ensure suitable matches. The Kaplan-Meier analysis was used to generate cumulative survival curves, and the log-rank test was used to compare the differences. Univariate and multivariate survival analyses were performed using Cox proportional hazards models, and hazard ratios (HRs) were computed with 95% confidence intervals (CIs). Fine-Gray competing-risk model was used to estimate the cumulative incidence of CSS by the “cmprsk” R package. Univariate and multivariate analysis were performed using the Fine-Gray competing-risk model to identify independent risk factors affecting CSS. The statistical analyses were performed using R software version 4.2.0. A p-value < 0.05 indicated statistical significance.

## 3 Results

### 3.1 Clinical basic characteristics

A total of 2,614 patients with HCC were extracted from the SEER database. 1105 were not treated with chemotherapy and/or radiotherapy and 1509 were treated with chemotherapy and/or radiotherapy, of which 1313 patients received chemotherapy or radiation therapy and 196 patients received both chemotherapy and radiotherapy. About 45.0% patients are > 62 years old and predominantly male. Among our subjects, the white population had a significantly higher incidence of HCC than other races. Interestingly, the majority of the unmarried are more inclined to do no treatment at all, while the married opted for chemotherapy or radiotherapy. Most patients were AFP positive and without lymph node and distant metastases. Of the patients under treatment, nearly 90% received chemotherapy or radiotherapy, and very few received both chemotherapy and radiotherapy. More details are shown in **Table 1**.

**Table 1:** Baseline characteristics of all eligible patients before and after matching.

Variables	Before PSM			After PSM		
	None (n, %)	Therapy (n, %)	P-value	None (n, %)	Therapy (n, %)	P-value
All	1105	1509		1070	1070	
Age,years			0.072			0.960
<54	207 (18.7)	259 (17.2)		197 (18.4)	192 (17.9)	
54-62	383 (34.7)	589 (39.0)		382 (35.7)	383 (35.8)	
>62	515 (46.6)	661 (43.8)		491 (45.9)	495 (46.3)	
Sex			0.506			0.533
Female	191 (17.3)	246 (16.3)		183 (17.1)	194 (18.1)	
Male	914 (82.7)	1263 (83.7)		887 (82.9)	876 (81.9)	
Race			0.273			0.289
White	743 (67.2)	983 (65.1)		709 (66.3)	702 (65.6)	
Black	157 (14.2)	249 (16.5)		156 (14.5)	180 (16.8)	
Other	205 (18.6)	277 (18.4)		205 (19.2)	188 (17.6)	
Marital status			<0.001			1
Unmarried	590 (53.4)	674 (44.7)		560 (52.3)	560 (52.3)	
Married	515 (46.6)	835 (55.3)		510 (47.7)	510 (47.7)	
AFP			0.808			0.945
Negative	132 (11.9)	185 (12.3)		119 (11.1)	120 (11.2)	
Positive	973 (88.1)	1324 (87.7)		951 (88.9)	950 (88.8)	
Tumor size,cm			0.079			0.924
<6.2	429 (38.8)	545 (36.1)		413 (38.6)	405 (37.9)	
6.2-9.3	263 (23.8)	417 (27.6)		255 (23.8)	255 (23.8)	
>9.3	413 (37.4)	547 (36.2)		402 (37.6)	410 (38.3)	
N stage			0.053			0.318
N0	932 (84.3)	1229 (81.4)		906 (84.7)	889 (83.1)	
N1	173 (15.7)	280 (18.6)		164 (15.3)	181 (16.9)	
M stage			0.922			0.632
M0	860 (77.8)	1172 (77.7)		842 (78.7)	851 (79.5)	
M1	245 (22.2)	337 (22.3)		228 (21.3)	219 (20.5)	
Therapy						
Chemotherapy or Radiotherapy		1313(87.0)				
Chemotherapy and Radiotherapy		196(13.0)				

**Figure 3:**
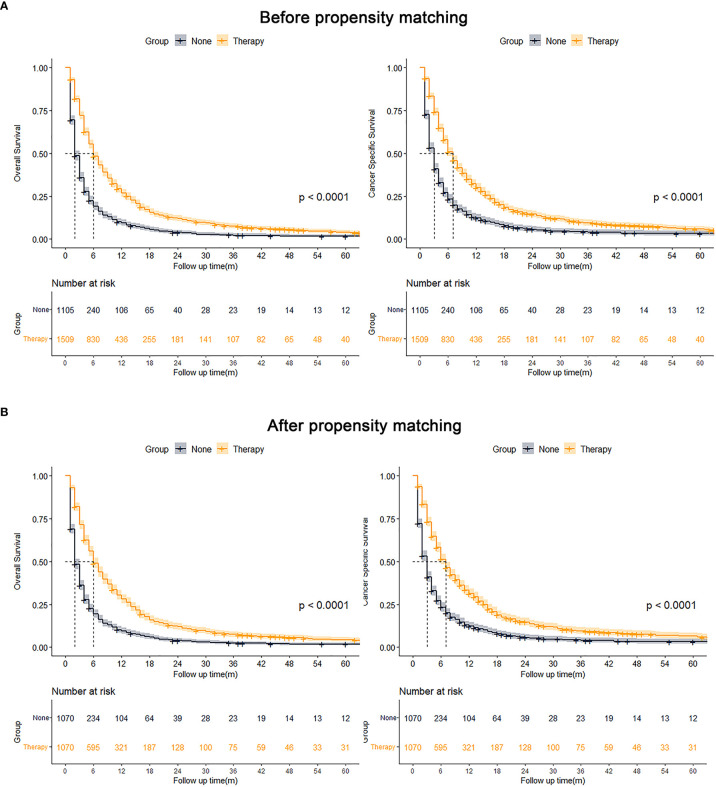
Kaplan-Meier analysis results for OS and CSS before (**A**, left, OS; right, CSS) and after (**B**, left, OS; right, CSS) PSM of all patients.

After grouping according to whether or not to receive treatment, using PSM for age, sex, race, marital status, AFP, tumor size, N stage, and M stage for 1:1 proximity matching, all variables in the two matched groups were not significantly different after PSM. The Kaplan-Meier analysis showed OS (p < 0.001) and CSS (p < 0.001) of the matched pre- and post-treatment groups were superior to the untreated group (**Figure 3**).

### 3.2 Survival analysis of all patients

Univariate and multivariate Cox analysis results of all patients before PSM showed AFP, tumor size, N stage, M stage, and treatment were considered as independent predictors of OS. Especially in terms of treatment modality, patients who received COR (HR = 0.538, 95% CI 0.495-0.585, p < 0.001) and CAR (HR = 0.371, 95% CI 0.316-0.436, p < 0.001) showed better OS compared to receive no treatment (**Table 2**). The same results are also shown after PSM (**Table 2**).

**Table 2:** Univariate and multivariate Cox analysis of OS for all eligible patients.

Variables	Before PSM						After PSM					
	Univariate			Multivariate			Univariate			Multivariate		
	HR	95% CI	P-value	HR	95% CI	P-value	HR	95% CI	P-value	HR	95% CI	P-value
Age,years												
<54	reference		0.910				reference		0.926			
54-62	1.010	0.902-1.131	0.865	–	–	–	1.018	0.899-1.153	0.781	–	–	–
>62	1.023	0.917-1.141	0.685	–	–	–	0.999	0.887-1.126	0.993	–	–	–
Sex												
Female	reference						reference					
Male	1.005	0.949-1.172	0.322	–	–	–	1.034	0.923-1.159	0.559	–	–	–
Race												
White	reference		0.391				reference		0.118			
Black	1.008	0.903-1.126	0.889	–	–	–	1.043	0.924-1.177	0.500	–	–	–
Other	1.075	0.969-1.193	0.173	–	–	–	1.128	1.005-1.265	0.040	–	–	–
Marital status												
Unmarried	reference						reference					
Married	0.954	0.882-1.032	0.241	–	–	–	0.995	0.913-1.085	0.917	–	–	–
AFP												
Negative	reference			reference			reference			reference		
Positive	1.423	1.260-1.606	<0.001	1.466	1.298-1.655	<0.001	1.395	1.214-1.602	< 0.001	1.437	1.250-1.652	< 0.001
Tumor size,cm												
<6.2	reference		<0.001	reference		<0.001	reference		< 0.001	reference		< 0.001
6.2-9.3	1.208	1.092-1.335	<0.001	1.289	1.165-1.245	<0.001	1.250	1.117-1.400	< 0.001	1.286	1.148-1.441	< 0.001
>9.3	1.450	1.323-1.589	<0.001	1.459	1.330-1.600	<0.001	1.415	1.281-1.564	< 0.001	1.425	1.289-1.576	< 0.001
N stage												
N0	reference		<0.001	reference		0.002	reference		< 0.001	reference		0.010
N1	1.255	1.132-1.392		1.179	1.060-1.311		1.280	1.138-1.439		1.171	1.039-1.321	
M stage												
M0	reference		<0.001	reference		<0.001	reference		< 0.001	reference		< 0.001
M1	1.157	1.428-1.725		1.482	1.344-1.633		1.608	1.445-1.789		1.473	1.320-1.644	
Therapy												
None	reference		<0.001	reference		<0.001	reference		< 0.001	reference		< 0.001
Chemotherapy or Radiotherapy	0.549	0.505-0.596	<0.001	0.538	0.495-0.585	<0.001	0.550	0.502-0.602	< 0.001	0.546	0.498-0.598	< 0.001
Chemotherapy and Radiotherapy	0.398	0.339-0.467	<0.001	0.371	0.316-0.436	<0.001	0.427	0.355-0.513	< 0.001	0.395	0.328-0.476	< 0.001

### 3.3 Survival analysis of patients receiving treatment

**Figure 4:**
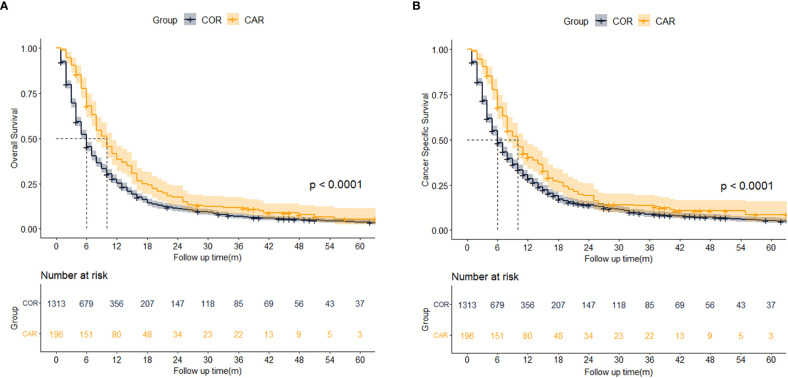
Kaplan-Meier analysis results for OS **(A)** and CSS **(B)** between COR and CAR. COR (Blue): Chemotherapy or Radiotherapy; CAR (Yellow): Chemotherapy and Radiotherapy.

We further explored the impact of different treatment strategies (COR and CAR) on patient survival. There were no significant differences between the COR and CAR groups, so PSM matching was not needed (**Table 3**). Kaplan-Meier analysis showed CAR had better OS (10 vs. 5 months, p <0.001) (**Figure 4A**) and CSS (10 vs. 6 months, p <0.001) (**Figure 4B**) than the COR. The 1-, 3-, and 5-year cumulative cancer-specific death risks were 62.9%, 89.6%, and 93.4% in the CAR group, compared with 75.3%, 93.5%, and 96.4% in the COR group. (**Figure 4**).

**Table 3:** Characteristics of COR and CAR patients.

Variables	All N=1509	COR group N=1313	CAR group N=196	P-value
Age,years				0.682
<54	259 (17.2)	229 (17.4)	30 (15.3)	
54-62	589 (39.0)	508 (38.7)	81 (41.3)	
>62	661 (43.8)	576 (43.9)	85 (43.4)	
Sex				0.671
Female	246 (16.3)	212 (16.1)	34 (17.3)	
Male	1263 (83.7)	1101 (83.9)	162 (82.7)	
Race				0.471
White	983 (65.1)	849 (64.7)	134 (68.4)	
Black	249 (16.5)	217 (16.5)	32 (16.3)	
Other	277 (18.4)	247 (18.8)	30 (15.3)	
Marital status				0.300
Unmarried	674 (44.7)	593 (45.2)	81 (41.3)	
Married	835 (55.3)	720 (54.8)	115 (58.7)	
AFP				0.645
Negative	185 (12.3)	159 (12.1)	26 (13.3)	
Positive	1324 (87.7)	1154 (87.9)	170 (86.7)	
Tumor size,cm				0.356
<6.2	545 (36.1)	473 (36.0)	72 (36.7)	
6.2-9.3	417 (27.6)	356 (27.1)	61 (31.1)	
>9.3	547 (36.2)	484 (36.9)	63 (32.1)	
N stage				0.362
N0	1229 (81.4)	1074 (81.8)	155 (79.1)	
N1	280 (18.6)	239 (18.2)	41 (20.9)	
M stage				0.090
M0	1172 (77.7)	1029 (78.4)	143 (73.0)	
M1	337 (22.3)	284 (21.6)	53 (27.0)	

Univariate and multivariate Cox analysis results showed AFP (HR = 1.454, 95% CI 1.241-1.74, p < 0.001), N stage (HR = 1.195, 95% CI 1.041-1.372, p = 0.012) M stage (HR = 1.797, 95% CI 1.577-2.048, p < 0.001) were independent risk factors for OS. Compared with tumor size < 6.2 cm, 6.2-9.3 cm (HR = 1.265, 95% CI 1.110-1.442, p < 0.001) and > 9.3 cm (HR = 1.528, 95% CI 1.351-1.729, p < 0.001) had worse OS. Patients who received CAR had better OS than COR. (HR = 0.657, 95% CI 0.561-0.769, p < 0.001) (**Table 4**). Univariate and multivariate competing-risk analysis results showed AFP (HR = 1.445, 95% CI 1.243-1.680, p < 0.001), M stage (HR = 1.623, 95% CI 1.404-1.877, p < 0.001) were independent risk factors for CSS. Compared with tumor size < 6.2 cm, tumor size > 9.3 cm (HR = 1.522, 95% CI 1.340-1.729, p < 0.001) had worse CSS. Patients who received CAR had better CSS than COR. (HR = 0.786, 95% CI 0.688-0.897, p < 0.001) (**Table 4**).

**Table 4:** Univariate and multivariate Cox analysis of OS and Competing-risk analysis for all eligible patients.

Variables	OS						CSS			
	Univariate			Multivariate			Univariate	Multivariate		
	HR	95% CI	P-value	HR	95% CI	P-value	P-value	HR	95% CI	P-value
Age,years										
<54	reference		0.878							
54-62	1.026	0.883-1.193	0.736	–	–	–				
>62	1.039	0.896-1.205	0.611	–	–	–	0.351	–	–	–
Sex										
Female	reference									
Male	1.148	0.998-1.322	0.054	–	–	–	0.197	–	–	–
Race										
White	reference		0.385							
Black	0.980	0.849-1.132	0.783	–	–	–				
Other	1.093	0.953-1.253	0.205	–	–	–	0.488	–	–	–
Marital status										
Unmarried	reference									
Married	0.997	0.898-1.106	0.949	–	–	–	0.057	–	–	–
AFP										
Negative	reference			reference				reference		
Positive	1.443	1.232-1.691	< 0.001	1.454	1.241-1.74	< 0.001	< 0.001	1.445	1.243-1.680	< 0.001
Tumor size,cm										
<6.2	reference		< 0.001	reference		< 0.001		reference		
6.2-9.3	1.241	1.088-1.414	0.001	1.265	1.110-1.442	< 0.001		1.123	0.982-1.283	0.089
>9.3	1.582	1.399-1.789	< 0.001	1.528	1.351-1.729	< 0.001	< 0.001	1.522	1.340-1.729	< 0.001
N stage										
N0	reference			reference				reference		
N1	1.356	1.187-1.549	< 0.001	1.195	1.041-1.372	0.012	< 0.001	1.077	0.925-1.255	0.340
M stage										
M0	reference			reference				reference		
M1	1.879	1.658-2.128	< 0.001	1.797	1.577-2.048	< 0.001	< 0.001	1.623	1.404-1.877	< 0.001
Therapy										
Chemotherapy or Radiotherapy	reference			reference				reference		
Chemotherapy and Radiotherapy	0.706	0.604-0.826	< 0.001	0.657	0.561-0.769	< 0.001	0.019	0.786	0.688-0.897	< 0.001

### 3.4 Survival analysis of different treatment strategies

To identify the effect of various factors on different treatment groups, univariate and multivariate analyses of OS and CSS were performed in each group. In the COR group, univariate and multivariate Cox analysis results showed AFP (HR = 1.416, 95% CI 1.195-1.679, p < 0.001), tumor size (6.2-9.3 cm: HR = 1.258, 95% CI 1.093-1.448, p = 0.001; > 9.3 cm HR = 1.602, 95% CI 1.405-1.827, p < 0.001), N stage (HR = 1.240, 95% CI 1.069-1.440, p = 0.005), M stage (HR = 1.786, 95% CI 1.550-2.058, p < 0.001) were independent risk factor of OS. Univariate and multivariate competing-risk analysis results showed AFP (HR = 1.420, 95% CI 1.205-1.670, p < 0.001), tumor size > 9.3 cm (HR = 1.520, 95% CI 1.330-1.740, p < 0.001), M stage (HR = 1.580, 95% CI 1.347-1.850, p = 0.005) were independent risk factor of CSS (**Table S1**). In the CAR group, AFP (HR = 1.765, 95% CI 1.132-2.752, p = 0.012) and M stage (HR = 1.693, 95% CI 1.205-2.379, p = 0.002) were independent risk factors of OS. Univariate and multivariate competing-risk analysis results showed M stage (HR = 2.038, 95% CI 1.486-2.790, p < 0.001) were independent risk factors of CSS (**Table S2**).

**Figure 5:**
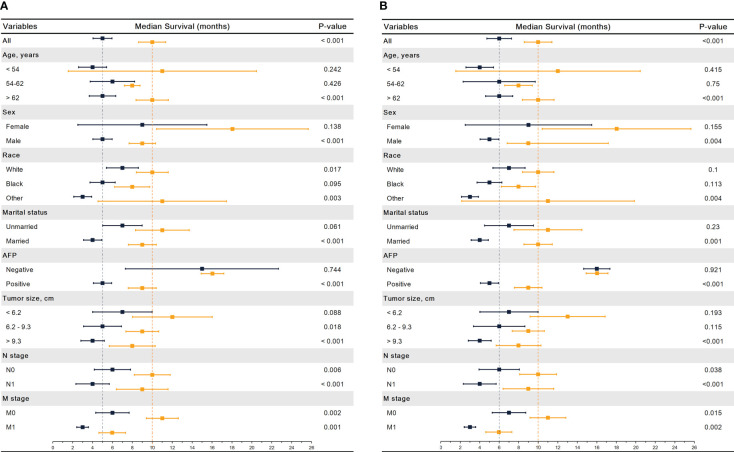
Median survival time of OS **(A)** and CSS **(B)** between COR and CAR in different subgroups. COR (Blue): Chemotherapy or Radiotherapy; CAR (Yellow): Chemotherapy and Radiotherapy.

When the two groups of patients were stratified by different variables, Kaplan-Meier analysis showed age > 62 (10.0 vs. 5.0 months, p<0.001), male (9.0 vs. 5.0 months, p<0.001), the white (10.0 vs. 7.0 months, p=0.017), other race (11.0 vs. 3.0 months, p = 0.003), married (9.0 vs. 9.0 months, p<0.001), AFP positive (9.0 vs. 5.0 months, p <0.001), tumor size > 9.3 cm (8.0 vs. 4.0 months, p<0.001)), N1 (9.0 vs. 4.0 months, p<0.001), N1 (10.0 vs. 6.0 months, p=0.006),M0 (11.0 vs. 6.0 months, p = 0.002), and M1 (6.0 vs. 6.0 months, p = 0.001) had better OS in the CAR group, while no significant differences were seen within the other groups (**Figure 5A**). The same result is also showed in CSS except for the white (**Figure 5B**).

## 4 Discussion

Macrovascular invasion has been recognized as one of the most important adverse prognostic factors affecting the long-term survival of patients with HCC (11). And most patients have already lost the chance of radical surgery by the time of diagnosis. Chemotherapy and radiotherapy techniques are now increasingly used for patients with unresectable PVTT/HVTT-HCC. However, there is a lack of multicenter, large-scale studies on the optimal choice of chemotherapy combined with radiotherapy versus mono-therapy, which makes it difficult to make clinical treatment decisions.

Traditionally, HVTT-HCC and PVTT-HCC have been treated with approximately the same strategy. Untreated patients with concomitant macrovascular invasion have a survival rate of just 2-4 months (12). In this study, when analysing all patients, both before and after PSM matching, it was found that patients who did not receive therapy had an OS of 2 months and a CSS of 3 months, while those who received chemotherapy and/or radiotherapy had an OS of 6 months and a CSS of 7 months, showing that chemotherapy and radiotherapy can significantly improve patients’ survival.

At present, chemotherapy modalities mainly include traditional systemic chemotherapy and hepatic artery infusion chemotherapy (HAIC). Traditional systemic chemotherapy is limited in clinical application due to severe systemic adverse reactions, while HAIC is widely used because it can carry anti-cancer drugs directly to the tumor site and reduce systemic adverse reactions. HAIC was first proposed by Japanese researchers and recommended by Japanese guidelines as the standard treatment for PVTT-HCC. Most of these HAIC-based studies focused on patients with PVTT-HCC, and 5-fluorouracil and systemic interferon or cisplatin were reported to be the most effective combination chemotherapy for PVTT-HCC with a median survival time of approximately 7 months (13, 14). The results of a phase III clinical study conducted by Lyu et al. showed that the objective response rate of the FOLFOX⁃HAIC protocol for the treatment of advanced HCC with high tumor burden (52.3% of patients had portal vein tumor thrombus) reached 31.5% (RECIST criteria), and 12.3% of the patients were successfully converted (15). Wu et al. investigated the effect of TACE combined HAIC versus TACE alone on patient survival and showed that the TACE combined HAIC had a longer median OS than the TACE group (P < 0.05) (16). Ahn YE et al. compared the effect of sorafenib and HAIC on survival in patients with PVTT and found no significant difference in OS (6.4 vs. 10.0 months, P = 0.139), but HAIC had a longer time to tumor progression (TTP) (6.2 vs. 2.1 months, P = 0.006) and higher disease control rate (DCR) (76% vs 37%, P = 0.001) (17).

Conventional radiotherapy is not suitable for HCC because the liver is a radiation-sensitive viscus and the lack of precise irradiation of the target area usually involves the surrounding normal liver tissue, leading to an increased incidence of radiation liver disease. However, with the advent of new radiation technologies such as 3-Dimensional Conformal Radiation Therapy(3D-CRT), Stereotactic Body Radiation Therapy (SBRT), and proton beam therapy (PBT), the clinical application of external radiotherapy for liver cancer is becoming more widespread. Previous studies reported that the OS of patients with PVTT/HVTT-HCC could be appropriately prolonged from 6 to 18 months regardless of radiotherapy modality (18–21). A retrospective study found that PBT improved local control and survival with PVTT-HCC, with a median progression-free survival time (PFS) of 2.3 years. Two of the patients lived 4.3 and 6.4 years, respectively, without relapse and severe adverse reactions (22). Shui et al. reported 70 patients with PVTT-HCC treated with SBRT. The median follow-up period was 9.5 months (range 1.0-21.0 months). Median survival time was 10.0 months (95%CI, 7.7-12.3 months) and overall survival rate (OS) at 6 and 12 months was 67.3% and 40.0%, Patients who respond well to radiation generally have better survival (23). The results of this study also found that either chemotherapy or radiotherapy prolonged patients’ OS from 2 to 6 months and CSS from 3 to 7 months, which is consistent with the results of previous studies.

For the treatment strategy of chemotherapy combined with radiotherapy, previous single-center, small sample size studies have found that HAIC combined with radiotherapy was effective in extending the OS of patients with PVTT from 7.5 months to 12 months (24–26). In a study with only a small sample size, the treatment efficiency of HVTT could be increased from 37% to 79% when HAIC was combined with radiotherapy (27). A Japanese study showed that surgical resection was safe and long-term beneficial for patients with PVTT after downstaging treatment though chemotherapy combined radiotherapy (28). In our study, chemotherapy combined with radiotherapy was found to be effective in prolonging patients’ OS (10 vs. 5 months, P < 0.001) and CSS (10 vs. 6 months, P < 0.001) compared to mono-therapy. It can be seen that this combined therapy strategy can better prolong the OS and CSS of patients and benefit them.

Next, we explored the prognostic factors affecting patients with concomitant PVTT/HVTT. In the survival analysis of all patients, we found that AFP, tumor size, N and M stage, and therapy strategy were independently associated with patient OS. To further explore the impact of different treatment strategies (COR and CAR) on patient survival. We used Cox proportional hazards model and competing-risk model to analyze OS and CSS in the whole treatment group and in each group, and found that AFP and distant metastasis were always the main independent factors affecting OS and CSS regardless of therapy strategy, which is consistent with previous findings (19, 25, 29, 30).

In 2017, based on the CheckMate 040 study, the FDA approved the PD-1 inhibitor (Nivolumab) for advanced HCC patients who did not respond to sorafenib therapy, marking the arrival of the era of HCC immunotherapy (31). Subsequently, the combination of PD-L1 inhibitor (atezolizumab) and vascular endothelial growth factor (VEGF) inhibitor (bevacizumab) proved to be the first drug with significantly better efficacy than sorafenib in more than a decade, ushering in a new era of combination therapy for HCC immunotherapy (5). At present, the main program of combined therapy with ICI is CTLA-4 inhibitor combined with PD-1/PD-L1 inhibitor. The current second-line regimen of nabuliumab in combination with CTLA-4 inhibitor (ipilimumab) is the best option, based on results from Cohort 4 in CheckMate 040. The combination regimen with the highest dose of ipilimumab showed the best efficacy, but also a higher incidence of adverse reactions (32).

The latest results of KEYNOTE-524, a phase I single-arm trial, showed that the mOS, mPFS and ORR of Lenvatinib combined with Pembrolizumab for advanced HCC were 22 months, 8.6 months and 36.0%, this combination regimen has been granted by the US FDA as a breakthrough therapy for the first-line treatment of unresectable HCC (33). In recent years, a number of ICI-based combination therapies have achieved good results in clinical trials, benefiting more patients with end-stage HCC, especially those with PVTT/HVTT.

Obviously, our study also has several limitations. First of all, this study is a retrospective study, and selection bias caused by incomplete data is inevitable. Although we adopted PSM to avoid selection bias, potential confounding factors cannot be ruled out. Second, because the SEER database only records yes or no for chemotherapy and radiotherapy, the lack of detailed information on chemotherapy and radiotherapy makes it difficult to conduct further studies, which also bodes well for more precise studies of the impact of each treatment on patient benefit. Undeniably, with the development of targeted and immunotherapy in recent years, the treatment of HCC has undergone earth-changing changes, greatly improving the survival of patients with advanced HCC. Unfortunately, the SEER database did not record information of targeted or immunotherapy for patients from 2004 to 2015. So we put it up in limitation; Finally, portal vein tumor thrombus and hepatic vein tumor thrombus were uniformly recorded in the SEER database as macrovascular invasion, which could not be studied separately. Even so, we demonstrated the benefit of radiotherapy combined with chemotherapy for long-term survival in PVTT/HVTT-HCC patients using a country-scale database.

## 5 Conclusion

It is recommended that surgically unresectable advanced PVTT/HVTT-HCC be treated with radiotherapy combined with chemotherapy. Positive AFP and distant metastasis are risk factors affecting patients’ long-term survival, and it is suggestive to actively treat these patients to prolong the survival of patients.

## Data availability statement

The datasets presented in this study can be found in online repositories. The names of the repository/repositories and accession number(s) can be found in the article/**Supplementary Material**.

## Ethics statement

Ethical review and approval was not required for the study on human participants in accordance with the local legislation and institutional requirements. Written informed consent for participation was not required for this study in accordance with the national legislation and the institutional requirements. Written informed consent was not obtained from the individual(s) for the publication of any potentially identifiable images or data included in this article.

## Author contributions

XQ, JC, and HC contributed to the conception and design of the study. JY, CX, RL, JX, JZha, XS, and TL collected the details of the patients and extracted and analyzed the data. XQ, JC, and HC drafted the manuscript. JZhe, YZ, and YY contributed with a critical revision of the manuscript. All authors contributed to the article and approved the submitted version.
