# Supplementary material for: Chemotherapy combined with radiotherapy can benefit more unresectable HCC patients with portal and/or hepatic vein invasion: a retrospective analysis of the SEER database
Source: Front Oncol. 2023 Jun 20;13:1098686. doi: 10.3389/fonc.2023.1098686 (PMC10319410; doi:10.3389/fonc.2023.1098686)
Supplement: Supplementary file 1 [file Table_1.docx]

| COR | OS | | | | | | CSS | | | |
| --- | --- | --- | --- | --- | --- | --- | --- | --- | --- | --- |
|  | Univariate | | | Multivariate | | | Univariate | Multivariate | | |
| Variables | HR | 95% CI | P-value | HR | 95% CI | P-value | P-value | HR | 95% CI | P-value |
| Age, years |  |  |  |  |  |  |  |  |  |  |
| <54 | reference |  | 0.654 | - |  |  |  |  |  |  |
| 54-62 | 1.038 | 0.884-1.219 | 0.652 | - | - | - |  |  |  |  |
| >62 | 1.074 | 0.917-1.258 | 0.375 | - | - | - | 0.763 | - | - | - |
| Sex |  |  |  |  |  |  |  |  |  |  |
| Female | reference |  |  |  |  |  |  |  |  |  |
| Male | 1.086 | 0.935-1.263 | 0.279 | - | - | - | 0.231 | - | - | - |
| Race |  |  |  |  |  |  |  |  |  |  |
| White | reference |  | 0.484 |  |  |  |  |  |  |  |
| Black | 1.017 | 0.873-1.185 | 0.825 | - | - | - |  |  |  |  |
| Other | 1.094 | 0.945-1.265 | 0.228 | - | - | - | 0.275 | - | - | - |
| Marital status |  |  |  |  |  |  |  |  |  |  |
| Unmarried | reference |  |  |  |  |  |  |  |  |  |
| Married | 0.997 | 0.892-1.114 | 0.955 | - | - | - | 0.899 | - | - | - |
| AFP |  |  |  |  |  |  |  |  |  |  |
| Negative | reference |  |  | reference |  |  |  | reference |  |  |
| Positive | 1.420 | 1.199-1.683 | <0.001 | 1.416 | 1.195-1.679 | <0.001 | < 0.001 | 1.420 | 1.205-1.670 | < 0.001 |
| Tumor size, cm |  |  |  |  |  |  |  |  |  |  |
| <6.2 | reference |  | <0.001 | reference |  | <0.001 |  | reference |  |  |
| 6.2-9.3 | 1.258 | 1.093-1.448 | 0.001 | 1.264 | 1.098-1.455 | 0.001 |  | 1.090 | 0.947-1.260 | 0.220 |
| >9.3 | 1.602 | 1.405-1.827 | <0.001 | 1.535 | 1.345-1.751 | <0.001 | < 0.001 | 1.520 | 1.330-1.740 | <0.001 |
| N stage |  |  |  |  |  |  |  |  |  |  |
| N0 | reference |  |  | reference |  |  |  | reference |  |  |
| N1 | 1.461 | 1.267-1.686 | <0.001 | 1.240 | 1.069-1.440 | 0.005 | < 0.001 | 0.090 | 0.925-1.290 | 0.300 |
| M stage |  |  |  |  |  |  |  |  |  |  |
| M0 | reference |  |  | reference |  |  |  | reference |  |  |
| M1 | 1.982 | 1.173-2.269 | <0.001 | 1.786 | 1.550-2.058 | <0.001 | < 0.001 | 1.580 | 1.347-1.850 | <0.001 |

Supplementary Material

**Supplemental Table 1** Univariate and Multivariate Cox analysis of OS and CSS for Chemotherapy or Radiotherapy patients.

**Supplemental Table 2** Univariate and Multivariate Cox analysis of OS and CSS for Chemotherapy and Radiotherapy patients.

| CAR | OS | | | | | | CSS | | | |
| --- | --- | --- | --- | --- | --- | --- | --- | --- | --- | --- |
|  | Univariate | | | Multivariate | | | Univariate | Multivariate | | |
| Variables | HR | 95% CI | P-value | HR | 95% CI | P-value | P-value | HR | 95% CI | P-value |
| Age, years |  |  |  |  |  |  |  |  |  |  |
| <54 | reference |  | 0.612 |  |  |  |  |  |  |  |
| 54-62 | 0.988 | 0.644-1.515 | 0.954 | - | - | - |  |  |  |  |
| >62 | 0.854 | 0.559-1.303 | 0.463 | - | - | - | 0.635 | - | - | - |
| Sex |  |  |  |  |  |  |  |  |  |  |
| Female | reference |  |  | reference |  |  |  |  |  |  |
| Male | 1.598 | 1.065-2.397 | 0.024 | 1.465 | 0.968-2.217 | 0.071 | 0.091 | - | - | - |
| Race |  |  |  |  |  |  |  |  |  |  |
| White | reference |  |  |  |  |  |  |  |  |  |
| Black | 0.740 | 0.484-1.133 |  | - | - | - |  |  |  |  |
| Other | 1.009 | 0.665-1.530 | 0.369 | - | - | - | 0.651 | - | - | - |
| Marital status |  |  |  |  |  |  |  |  |  |  |
| Unmarried | reference |  |  |  |  |  |  |  |  |  |
| Married | 1.037 | 0.770-1.397 | 0.810 | - | - | - | 0.438 | - | - | - |
| AFP |  |  |  |  |  |  |  |  |  |  |
| Negative | reference |  |  | reference |  |  |  |  |  |  |
| Positive | 1.586 | 1.022-2.461 | 0.040 | 1.765 | 1.132-2.752 | 0.012 | 0.084 | - | - | - |
| Tumor size, cm |  |  |  |  |  |  |  |  |  |  |
| <6.2 | reference |  | 0.168 |  |  |  |  |  |  |  |
| 6.2-9.3 | 1.202 | 0.838-1.724 | 0.317 | - | - | - |  |  |  |  |
| >9.3 | 1.410 | 0.987-2.016 | 0.059 | - | - | - | 0.060 | - | - | - |
| N stage |  |  |  |  |  |  |  |  |  |  |
| N0 | reference |  |  |  |  |  |  |  |  |  |
| N1 | 1.005 | 0.697-1.450 | 0.979 | - | - | - | 0.545 | - | - | - |
| M stage |  |  |  |  |  |  |  |  |  |  |
| M0 | reference |  |  | reference |  |  |  | reference |  |  |
| M1 | 1.695 | 1.219-2.357 | 0.002 | 1.693 | 1.205-2.379 | 0.002 | < 0.001 | 2.038 | 1.486-2.790 | < 0.001 |
